# Green Space Morphology and School Myopia in China

**DOI:** 10.1001/jamaophthalmol.2023.6015

**Published:** 2024-01-04

**Authors:** Yahan Yang, Huipeng Liao, Lanqin Zhao, Xun Wang, XiaoWei Yang, Xiaohu Ding, Xuelong Li, Zhiyu Jiang, Xingying Zhang, Qingling Zhang, Huagui He, Liang Guo, Hualiang Lin, Guanghui Dong, Bryan Spencer, Mingguang He, Nathan Congdon, Ian George Morgan, Haotian Lin

**Affiliations:** 1 State Key Laboratory of Ophthalmology, Zhongshan Ophthalmic Centre, Sun Yat-sen University, Guangdong Provincial Key Laboratory of Ophthalmology and Vision Science, Guangdong Provincial Clinical Research Center for Ocular Diseases, Guangzhou, China; 2 Department of Epidemiology, Gillings School of Global Public Health, University of North Carolina, Chapel Hill; 3 Guangzhou Urban Planning and Design Survey Research Institute, Guangzhou, China; 4 Centre for OPTical IMagery Analysis and Learning (OPTIMAL), Northwestern Polytechnical University, Xi’an, China; 5 Key Laboratory of Radiometric Calibration and Validation for Environmental Satellites (LRCVES/CMA), National Satellite Meteorological Center, China Meteorological Administration (NSMC/CMA), Beijing, China; 6 School of Aeronautics and Astronautics, Sun Yat-sen University, Shenzhen, China; 7 Department of Epidemiology, School of Public Health, Sun Yat-sen University, Guangzhou, China; 8 Department of Preventive Medicine, School of Public Health, Sun Yat-sen University, Guangzhou, China; 9 Department of Management, College of Business, City University of Hong Kong, Hong Kong, Special Administrative Region of China; 10 PolyU School of Optometry, Hong Kong Polytechnic University, Hong Kong, Special Administrative Region of China; 11 Centre for Public Health, Queen’s University Belfast, Belfast, United Kingdom; 12 Orbis International, New York, New York; 13 Research School of Biology, College of Medicine, Biology and Environment, Australian National University, Canberra, Australia; 14 Hainan Eye Hospital and Key Laboratory of Ophthalmology, Zhongshan Ophthalmic Center, Sun Yat-sen University, Haikou, China; 15 Centre for Precision Medicine, Department of Genetics and Biomedical Informatics, Zhongshan School of Medicine, Sun Yat-sen University, Guangzhou, China

## Abstract

**Importance:**

China has experienced both rapid urbanization and major increases in myopia prevalence. Previous studies suggest that green space exposure reduces the risk of myopia, but the association between myopia risk and specific geometry and distribution characteristics of green space has yet to be explored. These must be understood to craft effective interventions to reduce myopia.

**Objective:**

To evaluate the associations between myopia and specific green space morphology using novel quantitative data from high-resolution satellite imaging.

**Design, Setting, and Participants:**

This prospective cohort study included students grades 1 to 4 (aged 6 to 9 years) in Shenzhen, China. Baseline data were collected in 2016-2017, and students were followed up in 2018-2019. Data were analyzed from September 2020 to January 2022.

**Exposures:**

Eight landscape metrics were calculated using land cover data from high-resolution Gaofen-2 satellite images to measure area, aggregation, and shape of green space.

**Main Outcome and Measures:**

The 2-year cumulative change in myopia prevalence at each school and incidence of myopia at the student level after 2 years were calculated as main outcomes. The associations between landscape metrics and school myopia were assessed, controlling for geographical, demographic, and socioeconomic factors. Principal component analyses were performed to further assess the joint effect of landscape metrics at the school and individual level.

**Results:**

A total of 138 735 students were assessed at baseline. Higher proportion, aggregation, and better connectivity of green space were correlated with slower increases in myopia prevalence. In the principal component regression, a 1-unit increase in the myopia-related green space morphology index (the first principal component) was negatively associated with a 1.7% (95% CI, −2.7 to −0.6) decrease in myopia prevalence change at the school level (*P* = .002). At the individual level, a 1-unit increase in myopia-related green space morphology index was associated with a 9.8% (95% CI, 4.1 to 15.1) reduction in the risk of incident myopia (*P* < .001), and the association remained after further adjustment for outdoor time, screen time, reading time, and parental myopia (adjusted odds ratio, 0.88; 95% CI, 0.80 to 0.97; *P* = .009).

**Conclusions and Relevance:**

Structure of green space was associated with a decreased relative risk of myopia, which may provide guidance for construction and renovation of schools. Since risk estimates only indicate correlations rather than causation, further interventional studies are needed to assess the effect on school myopia of urban planning and environmental designs, especially size and aggregation metrics of green space, on school myopia.

## Key Points

**Question:**

What is the association between green space morphology and childhood myopia?

**Findings:**

In this cohort study including 138 735 students from grades 1 to 4, using high-resolution satellite images, the proportion, aggregation, and connectivity of green space were found to be associated with 2-year changes in school myopia rate. Principal component analysis further supported the finding that overall green space morphology is inversely associated with prevalent and incident myopia.

**Meaning:**

Careful planning of green space development may be a strategy for myopia prevention.

## Teaser

This cohort study evaluates the associations between myopia and specific green space morphology using novel quantitative data from high-resolution satellite imaging.

## Introduction

Myopia (ie, short-sightedness) is a common cause of vision impairment and has become a prominent global health concern.^1^ The number of individuals with myopia is projected to increase to nearly 5 billion by 2050, among whom 20% will have high myopia and be at greater risk of developing cataracts, myopic macular degeneration, staphyloma, retinal detachments, and glaucoma.^2^ This global burden is distributed unevenly, with higher prevalence of myopia in East Asia, particularly China.^3^ To mitigate the surge in prevalence of myopia, it is important to understand the role of environmental factors in delaying onset and slowing progression and to explore effective approaches suitable for large-scale myopia prevention, particularly among school-aged children.^4^

Urbanization has led to an increasing proportion of the global population dwelling in cities.^5^ Previous research suggests that urban-dwelling children have a higher prevalence of myopia than rural-dwelling children.^1,6^ Given that green space exposure at school is inversely associated with spectacle use,^7^ deliberate planning of green space may improve the visual health of school-going children.

Remote sensing technology has been used investigate the effects of environmental factors on disease distribution and underlying mechanisms owing to its unique advantages, including large coverage, consistency, and cost-effectiveness.^8^ Using remote sensing technology, our recent cohort study demonstrated that a 0.1-unit increase in normalized difference vegetation index (NDVI), which is widely used to reflect the greenness of an environment, was associated with a 27.9% reduction in myopia risk.^9^ However, green space with the same NDVI may have different functions and utility for health due to variations in morphology.^5,10^ Green space morphology has proven associations with population health outcomes in multiple organ systems, such as the heart, lungs, and immune system.^11,12^ Specific characteristics of green space morphology, such as a higher area proportion and better-connected landscape spatial patterns, may help protect myopia by promoting exposure to green space in outdoor lighting or encouraging children to focus on natural landscapes with higher spatial frequencies. However, to our knowledge, no study has yet elucidated how green space morphology is associated with prevalent or incident myopia.

To address this shortcoming, the current study is designed to investigate the association between green space morphology and myopia among school-going children. Our analysis of various landscape metrics will provide guidance for landscape designs, which can be tested prospectively for their impact on myopia.

## Methods

### Study Design and Population

A prospective cohort study, the Environmental Health and Myopia Prevention and Control Project, was launched in 2016 with the aim of evaluating the association of environmental factors with prevalent and incident myopia.^13^ The study was approved by ethics committees and the Internal Review Board of Zhongshan Ophthalmic Center, Sun Yat-sen University. Written informed consent was obtained from parents or guardians of all child participants, following the principles of the Declaration of Helsinki. None of the participants were offered compensation or incentives to participate. The design of the study has been reported previously in detail.^9^ In brief, this study involved all of the schools drawn from the largest administrative districts (Bao’an district) of Shenzhen, a subtropical Chinese metropolis, that agreed to participate. Two-year change in refraction was measured among all school-attending children in grades 1 to 4 (aged 6 to 9 years) at the 113 involved schools. The number of children enrolled at each school varied between 444 and 5066. Students were excluded from this study if they reported a history of optical measures (such as wearing rigid contact lenses, widely used in the prevention of myopia by temporarily flattening the anterior face of the cornea, reducing its refractive power) or of medical treatment (such as using atropine) or any condition besides refractive error that might affect their vision or visual development, such as amblyopia or diabetes.

**Figure 1.**
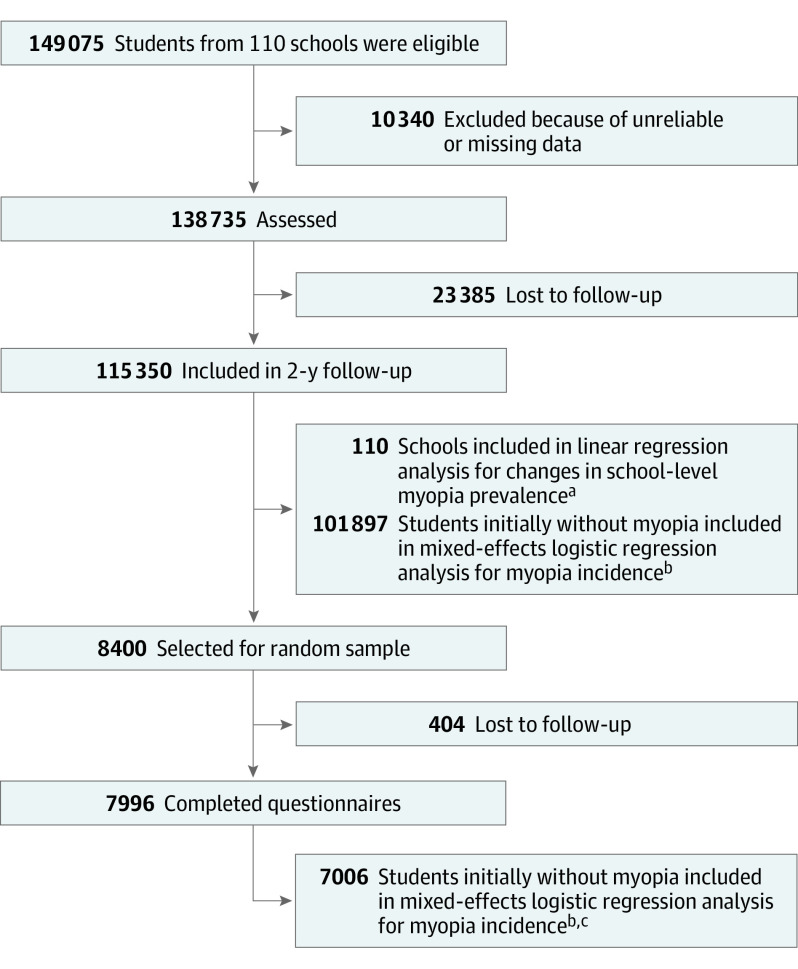
Overall Study Design and Participant Flow Diagram ^a^Linear regression was used for the school analysis and was adjusted for percentage of male students, mean age, myopia prevalence (ie, mean spherical equivalent refraction) at baseline, student density, and school status. ^b^Mixed-effects logistic regression was used for the student analysis and accounted for the school clustering, adjusting for sex, age, spherical equivalent refraction at baseline, student density, and school status. ^c^Additionally adjusted for paternal myopia, maternal myopia, mean screen time, mean reading time, and mean outdoor activity time after school per day.

Data from the above cohort of children were used in our study. Three schools were excluded due to their location in parks because their green space metrics’ values are outliers. In total, 110 schools were included in the analysis (Figure 1), with a mean (SD) of 1895 (416) students. The study followed the Strengthening the Reporting of Observational Studies in Epidemiology (STROBE) reporting guideline.

### Ocular Examinations

Ocular examinations were completed at the schools by 12 trained technicians. Visual acuity was assessed for each eye separately using tumbling E Early Treatment Diabetic Retinopathy Study (ETDRS) charts at a distance of 4 meters in a well-lit indoor area. Automated refraction without cycloplegia (used to control accommodation, the tendency to focus on near objects, which can interfere with accuracy of refraction) was performed separately in each eye using a Topcon RM6000 (Topcon). The spherical equivalent refraction (SER) was calculated as the spherical power plus 0.5 × cylindrical power. Similar procedures conducted by the same set of examiners with the same equipment were performed at annual follow-up examinations from 2016-2017 to 2018-2019, during the same month of the year at each school. The baseline data (2016-2017) and the year 2 (2018-2019) data were used for assessment of outcomes in the current study.

### Outcome Definition

Myopia was defined as SER of −0.5 diopters (D) or less.^14^ Data for the right eyes were used for analysis because the refractive powers in the right and left eyes of children were highly correlated. The myopia prevalence was defined at each school as the percentage of students with myopia. The school-level outcome was the 2-year change in myopia prevalence at each school, defined as a continuous variable. The individual-level outcome was the incidence of myopia at the student level in 2 years, defined as a binary variable.

### Measurement of Landscape Metrics

**Figure 2.**
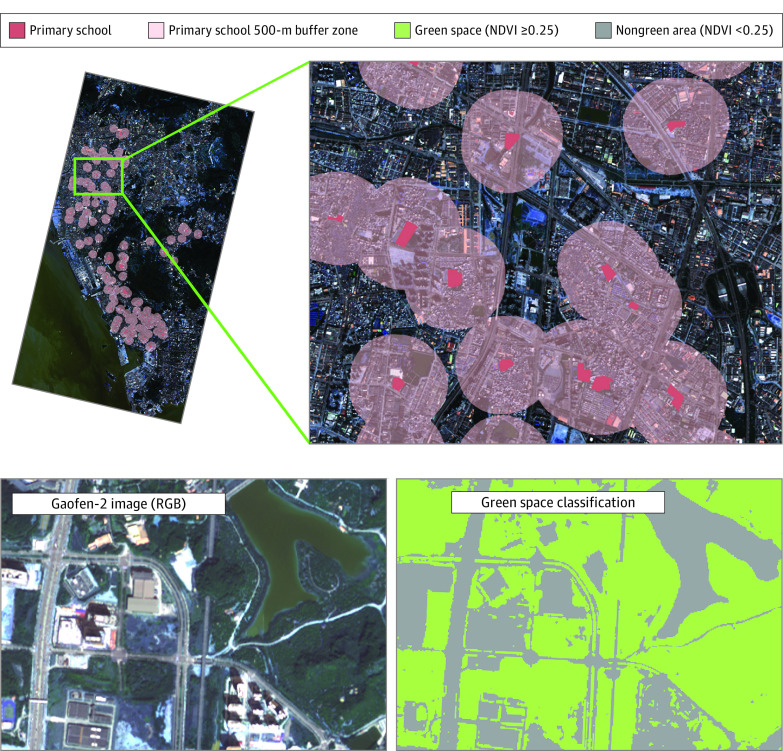
Campus Green Space Extraction and Spatial Metrics Calculation Using remote sensing images, school campuses (red) and surrounding 500-m buffer zones (pink) were assessed to calculate landscape metrics. For green space extraction, the threshold normalized difference vegetation index (NDVI) value for the classification of vegetation was set to 0.25, with higher values indicating green space (green) and lower values indicating nongreen area (gray). RGB indicates red, green, blue.

We focused on the green space morphology inside and around the school areas because Chinese students spend most of their day in schools (at least 9 hours in primary schools) including recess and physical education classes,^15^ and school-age children are enrolled in the schools near their residences.^16,17^ School campus areas were detected, and corresponding 500-meter buffer zones were created (eMethods in Supplement 1). The study used cloud-free Gaofen-2 satellite data obtained between 2016 and 2019 from the Resource and Environment Science and Data Center Data Cloud Platform.^18^ Images from February 15, 2017, and December 27, 2017, were selected for analysis after undergoing radiometric calibration, atmospheric correction, orthorectification, and mosaicking with ENVI software version 5.3 (Harris Corp). We used a widely used vegetation index, NDVI, to extract green space.^19^ It is calculated as NDVI = (NIR − R) /( NIR + R), where NIR is near-infrared (band 5) and R is red (band 4). The threshold for vegetation classification typically ranges between 0.2 and 0.3,^20,21,22,23^ and we used a threshold of 0.25 in our study (Figure 2).

Landscape metrics can be used to analyze the spatial geometry and distribution characteristics of green space. Eight landscape metrics were calculated using Fragstats version 4.2 (Kevin Mcgarigal and Eduard Ene): 3 size metrics, including percentage of landscape, area-weighted mean greenness area, and largest patch index; 4 aggregation metrics, including aggregation index, cohesion index, patch density, and area-weighted mean of proximity index; and 1 shape metric, area-weighted mean patch shape index.^24^ Definitions and detailed information of the landscape metrics were included in the eMethods in Supplement 1.

### Statistical Analysis

Normality was tested using the Shapiro-Wilk test and histogram. A log_10_ transformation was performed on nonnormally distributed data. Pairwise correlations for all green space metrics were performed using Pearson correlation analysis. Our hypothesis is that landscape metrics may affect the development of myopia, and we have therefore performed the association analysis at the 2-year follow-up stage. We investigated the association between each landscape metric and the change in school myopia prevalence. We also performed a principal component analysis to assess how landscape metrics as a whole affected school-level and individual-level outcomes. At the school level, we investigated the association between landscape structure and change in school-specific myopia rate using linear regression. In the regression analyses, 5 covariates were controlled for: percentage of boys in a school, mean age, myopia rate at baseline, total student density, and school socioeconomic ranking. To calculate school socioeconomic ranking, each school was comprehensively evaluated from 3 aspects: economic inputs and school infrastructure, educational outcomes and social impacts, and income. Total scores ranged from 0 to 500. Scores of more than 450 were considered high socioeconomic ranking, scores of 350 to 450 were considered moderate socioeconomic ranking, and scores less than 350 were considered low socioeconomic ranking. At the individual level, myopia incidence among students without myopia at baseline was calculated using SER. We investigated the association between landscape metrics and the incidence of myopia using mixed-effects logistic regression models controlling for the following covariates: sex, age, SER at baseline, total student density, and school socioeconomic status. To better estimate effects caused by potential confounding factors at the individual level, we randomly sampled 28 among the 110 schools and then sampled 25% of students per grade in each school, for a total subset of 8400 students. Based on the subset, student-level analyses were adjusted for 5 additional covariates, including paternal and maternal myopia, average screen time, reading time, and outdoor activity time after school per day. The details of the covariates are described in the eMethods in Supplement 1. Sensitivity analyses were conducted to assess alternate thresholds defining myopia (−0.75 D).

Each of the landscape metrics included in the regression models was examined against the others for collinearity. In all mixed-effects logistic regression models, a random intercept term was used to account for school clustering. All tests were 2-sided, and significance was set at *P* < .05; *P* values were not adjusted for multiple analyses. Data processing and statistical analyses were performed with R version 3.6.1 (The R Foundation) and SPSS version 24.0 (IBM).

## Results

From September 2016 to June 2017, a total of 149 075 grade 1 to 4 students underwent baseline assessment at the 110 participating schools in Shenzhen, China. Students who had unreliable or missing data were excluded. Thereafter, 138 735 students (93.1%) were assessed in the baseline analyses from September 2016 to June 2017. Among them, 115 350 (83.1%) were followed up 2 years later from September 2018 to June 2019. The mean (SD) increase in myopia prevalence was 21.2% (5.6%). Of the 101 897 students without myopia at baseline, 26 292 (25.8%) developed myopia during the follow-up period. The characteristics of the landscape metrics, myopia variables, and demographic variables are provided in eTable 1 in Supplement 1. The study included 21 schools in the highest socioeconomic ranking (19.1%), 52 schools in the moderate socioeconomic ranking (47.3%), and 37 schools in the low socio-economic ranking (33.6%) at baseline. The associations between landscape metrics are provided in eFigure 1 in Supplement 1.

**Figure 3.**
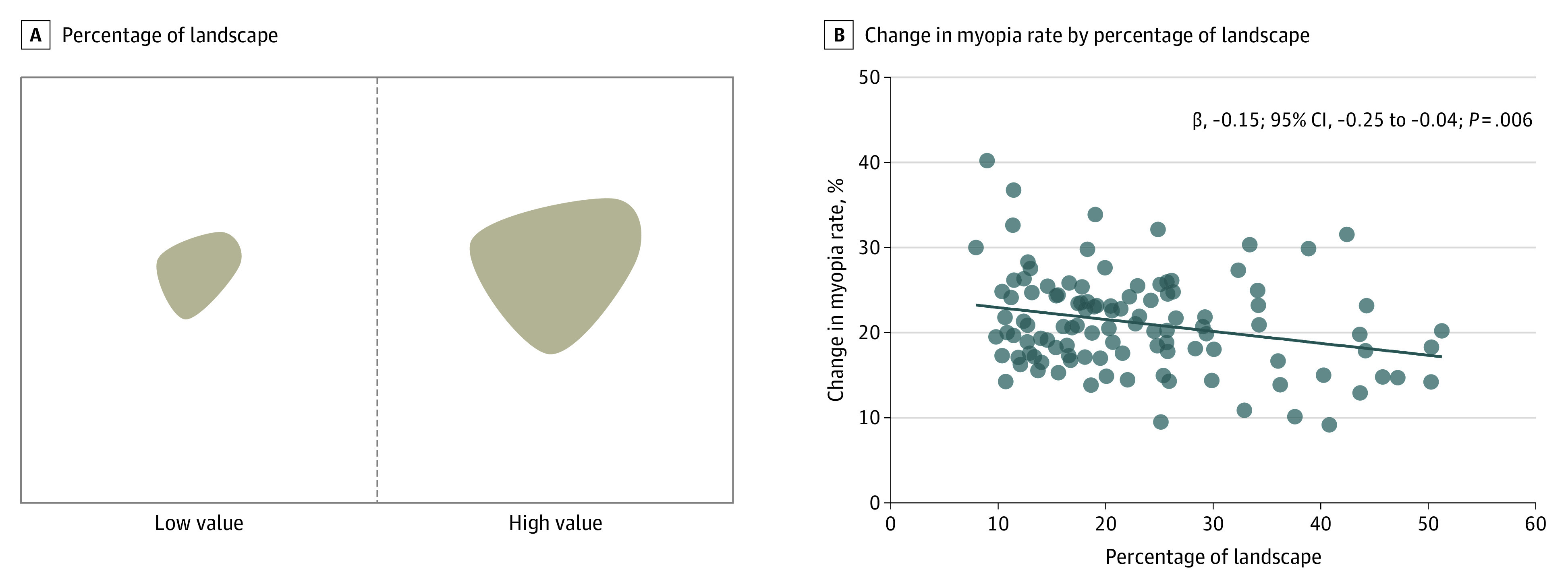
Association Between Myopia and Percentage of Landscape A, A higher value of percentage of landscape indicates a higher ratio of green space over the total area. B, With a 10% increase in percentage of landscape, the change in myopia rate decreased by 1.5% (95% CI, −2.5 to −0.4; *P* = .006).

**Figure 4.**
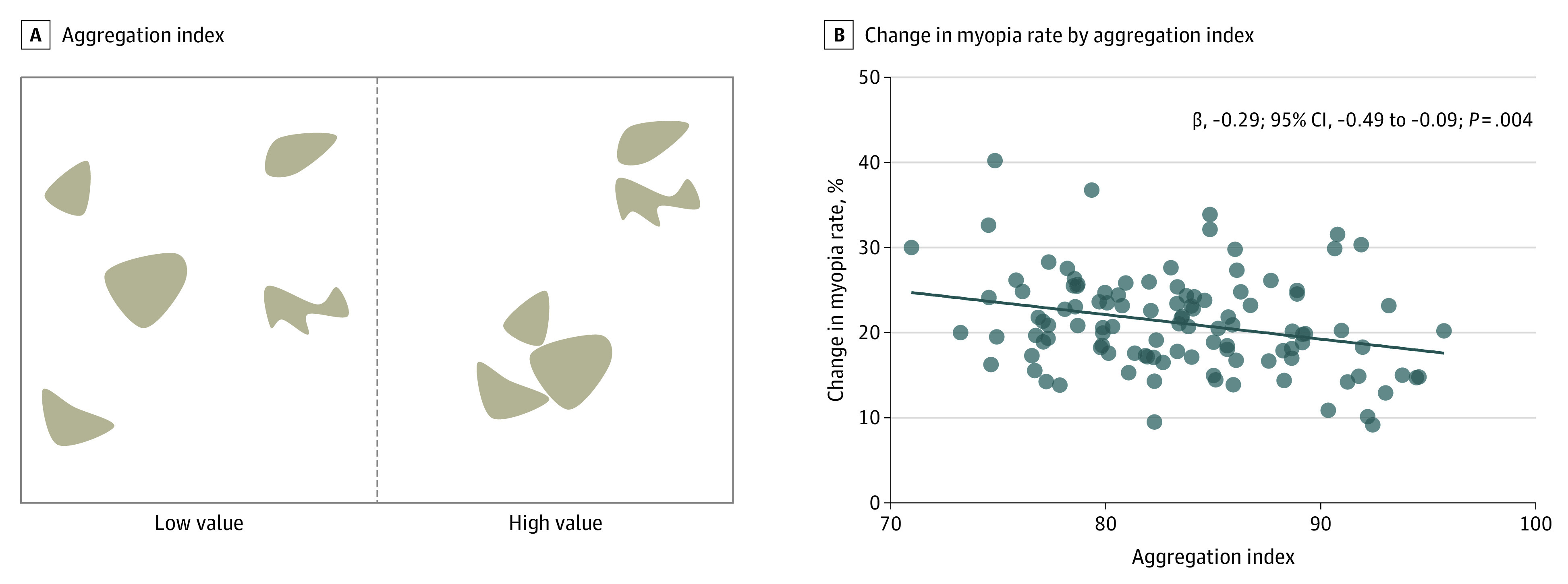
Association Between Myopia and Aggregation Index A, A higher value of aggregation index indicates a more aggregated green space pattern. B, Higher aggregation index led to a reduction in myopia rate change (−0.3%; 95% CI, −0.5 to −0.1; *P* = .004).

With a 10% increase in the percentage of landscape, the 2-year change in myopia prevalence was 1.5% (95% CI, −2.5 to −0.4; *P* = .006) lower (Figure 3). A higher aggregation index was associated with a reduced change in myopia rate (−0.3%; 95% CI, −0.5 to −0.1; *P* = .004; Figure 4). Furthermore, increased cohesion index was associated with a slower increase in school myopia rate (−0.5%; 95% CI, −0.8 to −0.2; *P* = .004; eFigure 2 in Supplement 1). Sensitivity analyses assessing alternate thresholds defining myopia had similar results (eTable 2 in Supplement 1). The associations between other metrics and myopia are shown in the eFigures 3 to 7 in Supplement 1.

**Figure 5.**
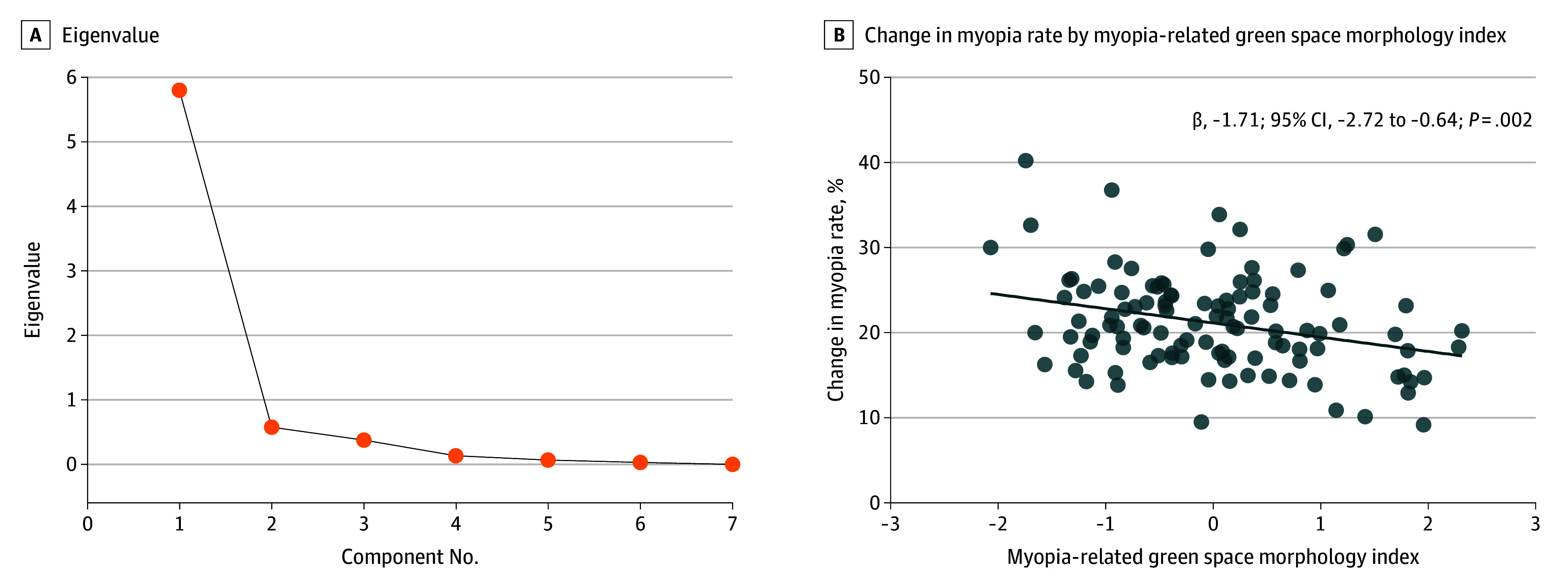
Principal Component Regression A, The first principal component, which included the contributions from the 7 landscape metrics that were associated with myopia (ie, myopia-related green space morphology index), was the only component with an eigenvalue larger than 1 and accounted for 82.9% of the total variance. A 1-unit increase in the myopia-related green space morphology index indicates 1 SD higher in proportion of the total and largest green space patches, larger patch sizes, increased aggregation, improved connectivity, greater segmentation of green space patches, and shorter in distances between the patches. B, With the myopia-related green space morphology index increased, we observed a decrease in the changes in school myopia rate (−1.7%; 95% CI, −2.7 to −0.6; *P* = .002).

The first principal component, which included contributions from all landscape metrics that were associated with myopia, defined as myopia-related green space morphology index here, was the only component with an eigenvalue greater than 1 (5.799) and accounted for 82.9% of the total variance (Figure 5A). The absolute value of each landscape metric’s factor loading ranged from 0.73 to 0.88, showing a relatively uniform contribution to the component. A 1-unit increase in the myopia-related green space morphology index indicates 1 SD higher in proportion of the total and largest green space patches, larger patch sizes, increased aggregation, improved connectivity, greater segmentation of green space patches, and shorter in distances between the patches.

At the school level, an increase in myopia-related green space morphology index was associated with a smaller change in school myopia prevalence (−1.7%; 95% CI, −2.7 to −0.6; *P* = .002; Figure 5B; eTable 3 in Supplement 1). At the individual level, students without myopia on baseline refraction were selected to assess the association between green space morphology and myopia incidence, with adjustment for age, sex, baseline SER, total student density, and school socioeconomic status. An increase in myopia-related green space morphology index was associated with a 9.8% (95% CI, 4.1 to 15.1; *P* < .001; eTable 4 in Supplement 1) reduction in the risk of myopia incidence among students without myopia at baseline.

Among the random sample of 8400 students, a total of 7996 (95.2%) provided complete data, 7006 (87.6%) of whom did not have myopia at baseline. Adjusting for individual-level factors including parental myopia and daily time spent on reading, screen use, and outdoor activity after school, an increase in myopia-related green space morphology index was associated with a 11.8% (95% CI, 3.1 to 19.6; *P* = .009; eTable 5 in Supplement 1) reduction in the risk of incident myopia in students without baseline myopia.

## Discussion

Analyzing prospective cohort data on myopia and landscape metrics, calculated using high-resolution satellite images, we found a reduced risk of myopia among primary school students studying at school campuses with a larger greenness proportion, larger areas of green space, better connectivity between green patches, more aggregated green space, less fragmented green space, and shorter distance between patches. Principal component analysis of 7 relevant landscape metrics demonstrated an association with smaller increases in school myopia prevalence and incident myopia at the individual level, suggesting that the landscape structure as a whole was negatively associated with myopia development. Following our previous report on the association between mean NDVI and slower myopia progression, we believe that these underlying green space morphological characteristics, which were not captured in previous studies, could be informative for population-based urban planning strategies to prevent myopia.

It has been suggested that high myopia prevalence among urban-dwelling children may be associated with reduced access to green space.^6,8^ However, quantitative assessment of green space is challenging, so few prior reports have evaluated such relationships. Over the past decade, satellite technology has offered new insights into the discipline of epidemiology and its application to human health.^8^ An increasing number of health studies have used remotely sensed data to investigate the long-term effects of environmental factors on health-related states, including the effects of green space on risk for cardiovascular diseases and mental health disorders.^25,26^

Recent studies have also begun to explore the relationship between myopia and green space. In 2019, self-reported data on 2727 school children from a Barcelona cohort showed that students at schools with higher NDVI had lower spectacle use, after adjusting for sex, race and ethnicity, preterm birth, screen time, exposure to environmental tobacco smoke, and socioeconomic status.^7^ Peng et al^27^ reported an inverse association between NDVI and myopia prevalence among adolescents aged 15 to 19 years using study data obtained from a systematic review and meta-analysis by Holden et al,^2^ which was adjusted for research year and type of area. In 2021, based on objective clinical measures of myopia and high-resolution satellite data, our prospective study covering more than 100 000 schoolchildren also observed that a 0.1 increase in green space exposure (on a scale of 0 to 1) was associated with a 27.9% reduction in incident myopia after adjusting for age, sex, parental myopia, screen time, reading hours, outdoor activity time, and socioeconomic status.^9^ Preschool myopia has also been found to be associated with reduction of green space in Shenzhen adjusted for child age and sex, parental vision status, screen time, parental education, and income.^28^ However, previous studies focusing on myopia and green space have used NDVI, a very general variable, as an indicator to describe overall greenness. Specific indices, including size, shape, and distribution of green space, are easier to understand and have more concrete design implications. A better understanding of the relationship between green space morphology and myopia is not only valuable for health, education, and urban planning but can also provide a blueprint for creating a sustainable environment by optimizing the application of land use.

Analysis of various metrics indicates that both higher proportion and higher aggregation of green space are potential protective factors against myopia. By creating a more inviting environment, these factors may provide greater incentives for exposure to outdoor light, proven to protect against the development of myopia.^29,30^ Furthermore, a higher proportion of green space might also encourage children remaining indoors to focus on distant targets and reduce persistent and intense near work. Continuous near-work is well understood as a risk factor for myopia.^31^ It is also reported that natural outdoor environments, such as green space, typically have a higher frequency component than indoor and man-made outdoor environments, such as buildings, which have spatial frequency characteristics similar to those known to induce form-deprivation myopia in animal models.^32^ In addition, our results suggest that more connected green space, such as greenways, might increase the accessibility of green space and provide a more effective myopia control option.

A more nuanced understanding of the association between green space morphology and myopia will be important for developing practical myopia prevention strategies. Except for increased outdoor time, most current preventive strategies for myopia, such as the use of orthokeratology lens and low-dose atropine eye drops, require compliance at the individual level and are expensive.^33^ However, population-based preventive strategies, especially those addressing environmental risk factors, are more likely to be feasible, sustainable, and scalable in a cost-effective manner.^34^ Therefore, it is necessary to focus on better understanding environmental risk factors and geographical exposures, providing the kind of specific evidence that can support data-driven manipulation of green space.^8^ Our study suggests that optimizing green space morphology might lead to a decrease in the current heavy burden of myopia. These findings could be tested prospectively as part of future urban landscape designs, especially in regions with high demand for urban expansion or renewal, which often overlap with areas of high myopia prevalence in Asia.

### Strengths and Limitations

Strengths of the current study include (1) the use of high-resolution remote sensing images to improve the accuracy on the estimation of green space morphology; (2) the analysis of several specific parameters to describe various aspects of green space morphology; and (3) the study of a large population recruited in China, a setting where interventions to reduce the current myopia burden are highly relevant and the results can be generalized to a wider range of children.

Our study also has several limitations. First, student participants underwent refractive error measurement using noncycloplegic autorefraction. Although noncycloplegic autorefraction has acceptable sensitivity and specificity, at 88.6% and 86.1%, respectively, it can lead to the overestimation of myopia due to instrument accommodation.^35,36^ However, since all participants were evaluated at all time points in the same fashion, the likely effect will be to artificially and modestly inflate myopia incidence in a general way rather than differentially impacting observed associations between green space and myopia progression from baseline. Second, our risk estimates only indicate correlations. Interventional studies are still needed to investigate causation, that is, the effectiveness of manipulating green space morphology as a population strategy for myopia prevention. Third, although we used high-resolution remote sensing images (Gaofen-2 satellites with 4-day temporal resolution) to generate NDVIs, only 2 cloud-free scenes were available during the study period. Nonetheless, the effect of these limitations is minor because Shenzhen is a subtropical city with high evergreen vegetation cover,^37^ and the landscape metrics in Shenzhen remained relatively stable over the 2 years.^38^ Fourth, we analyzed 8 green space morphology indicators, which may increase the probability of false positives. Fifth, given that Shenzhen is a typical city with rapid urbanization, remarkable economic growth, and rising health concerns,^39^ the findings may not be generalizable to many parts of China and the global context, particularly in rural areas. However, they may still offer some insights for large cities in China and well-urbanized regions in Asia.

## Conclusions

In conclusion, this study provides evidence at the geographical level that specific parameters describing green space morphology are associated with development of myopia. A well-arranged green space with larger areas, better connectivity, increased aggregation, lower fragmentation, and shorter distance between patches was correlated with slower progression in school myopia prevalence. Prospective interventional studies are needed to assess the effects on childhood myopia of the intentional manipulation of green space layouts.

## Notes

Supplement 1.
eMethods.
**eFigure 1.** Heatmap of the Correlation Matrix of Landscape Metrics **eFigure 2.** Association Between Myopia and Largest Patch Index (LPI) **eFigure 3.** Association Between Myopia and Area-Weighted Mean of Greenness Area (AREA_AM) **eFigure 4.** Association Between Myopia and Area-Weighted Mean Shape Index (SHAPE_AM) **eFigure 5.** Association Between Myopia and Cohesion Index (COHESION) **eFigure 6.** Association Between Myopia and Patch Density (PD) **eFigure 7.** Association Between Myopia and Proximity Index (PROX_AM) **eTable 1.** Characteristics of the Landscape Metrics and Population of the Studied Schools **eTable 2.** Sensitivity Analyses of the Association Between Myopia-Related Green Space Morphology Index and School-Level Increase in Myopia Rate Using the Linear Regression Model **eTable 3.** Association Between Myopia-Related Green Space Morphology Index and School-Level Increase in Myopia Rate Using the Linear Regression Model **eTable 4.** Association Between Myopia-Related Green Space Morphology Index and Myopia Incidence at the Individual-Level Among Students Without Myopia Who Were Followed Using the Mixed-Effects Logistic Regression Model **eTable 5.** Association Between Myopia-Related Green Space Morphology Index and Myopia Incidence at the Individual-Level Among the Subset Without Myopia Who Completed Questionnaire Using the Mixed-Effects Logistic Regression Model
eReferences.

Supplement 2. Data Sharing Statement
