# Supplementary material for: Green Space Morphology and School Myopia in China
Source: JAMA Ophthalmol. 2024 Jan 4;142(2):115–22. doi: 10.1001/jamaophthalmol.2023.6015 (PMC10767644; doi:10.1001/jamaophthalmol.2023.6015)
Supplement: Supplement 1. — eMethods. eFigure 1. Heatmap of the Correlation Matrix of Landscape Metrics eFigure 2. Association Between Myopia and Largest Patch Index (LPI) eFigure 3. Association Between Myopia and Area-Weighted Mean of Greenness Area (AREA_AM) eFigure 4. Association Between Myopia and Area-Weighted Mean Shape Index (SHAPE_AM) eFigure 5. Association Between Myopia and Cohesion Index (COHESION) eFigure 6. Association Between Myopia and Patch Density (PD) eFigure 7. Association Between Myopia and Proximity Index (PROX_AM) eTable 1. Characteristics of the Landscape Metrics and Population of the Studied Schools eTable 2. Sensitivity Analyses of the Association Between Myopia-Related Green Space Morphology Index and School-Level Increase in Myopia Rate Using the Linear Regression Model eTable 3. Association Between Myopia-Related Green Space Morphology Index and School-Level Increase in Myopia Rate Using the Linear Regression Model eTable 4. Association Between Myopia-Related Green Space Morphology Index and Myopia Incidence at the Individual-Level Among Students Without Myopia Who Were Followed Using the Mixed-Effects Logistic Regression Model eTable 5. Association Between Myopia-Related Green Space Morphology Index and Myopia Incidence at the Individual-Level Among the Subset Without Myopia Who Completed Questionnaire Using the Mixed-Effects Logistic Regression Model eReferences. [file jamaophthalmol-e236015-s001.pdf]

Supplemental Online Content

Yang Y, Liao H, Zhao L, et al. Green space morphology and school myopia in China. *JAMA Ophthalmol*. Published online January 4, 2024.
doi:10.1001/jamaophthalmol.2023.6015

eMethods.
eFigure 1. Heatmap of the Correlation Matrix of Landscape Metrics
eFigure 2. Association Between Myopia and Largest Patch Index (LPI)
eFigure 3. Association Between Myopia and Area-Weighted Mean of Greenness Area (AREA_AM)
eFigure 4. Association Between Myopia and Area-Weighted Mean Shape Index (SHAPE_AM)
eFigure 5. Association Between Myopia and Cohesion Index (COHESION)
eFigure 6. Association Between Myopia and Patch Density (PD)
eFigure 7. Association Between Myopia and Proximity Index (PROX_AM)
eTable 1. Characteristics of the Landscape Metrics and Population of the Studied Schools
eTable 2. Sensitivity Analyses of the Association Between Myopia-Related Green Space Morphology Index and School-Level Increase in Myopia Rate Using the Linear Regression Model
eTable 3. Association Between Myopia-Related Green Space Morphology Index and School-Level Increase in Myopia Rate Using the Linear Regression Model
eTable 4. Association Between Myopia-Related Green Space Morphology Index and Myopia Incidence at the Individual-Level Among Students Without Myopia Who Were Followed Using the Mixed-Effects Logistic Regression Model
eTable 5. Association Between Myopia-Related Green Space Morphology Index and Myopia Incidence at the Individual-Level Among the Subset Without Myopia Who Completed Questionnaire Using the Mixed-Effects Logistic Regression Model
eReferences.

This supplementary material has been provided by the authors to give readers additional information about their work.

© 2024 Yang Y et al. *JAMA Ophthalmology*.

eMethods.

1. Campus area detection and buffer zone creation

The boundary of each school campus was manually sketched from the satellite images, and the school area was automatically calculated by ArcGIS (version 10.6, Esri, USA). We included the 110 school campuses (a mean area of 21,800 m^2) and surrounding 500-meter buffer zones (an approximately 7-minute walking distance,[1] extracted by FME [Feature Manipulate Engine, Safe Software, Canada)], for the calculation of landscape metrics.

2. Definitions of Landscape metrics

Index	Definition	Range
Size metrics:		
Percentage of landscape (PLAND)	Ratio of green space over the total area	0%<PLAND≤100%
Area-weighted mean greenness area (AREA_AM)	The mean of green patches with the consideration of their proportional abundance	AREA_AM≥0
Largest patch index (LPI)	Percentage occupied by the largest patch of green space out of a campus area	0%<LPI≤100%
Shape metric:		
Area-weighted mean patch shape index (SHAPE_AM)	Perimeter-area relationships to reflect the complexity of green patch shape in average, by considering patches' proportional abundance	SHAPE_AM>0
Aggregation metrics:		
Cohesion index (COHESION)	The physical connectivity of green patches in a campus	0<COHESION<100
Aggregation index (AI)	Like adjacencies of green space	0%≤AI≤100%
patch density (PD)	The number of green patches divided by campus area	PD≥0
Area-weighted mean of proximity index (PROX_AM)	The area and distance of green patches in a search radius of 500 meters to reflect the size and proximity of green patches in average, by considering patches' proportional abundance.	PROX_AM≥0

© 2024 Yang Y et al. *JAMA Ophthalmology.*

3. Other covariates

Potential confounding factors identified by previous studies, including sociodemographic data, age (of each participant and mean age at each school) and sex (of each participant and percentage of boys at each school),[2] was obtained from school records and included in our regression models. Student density was defined as the number of students divided by the total land area of each school in meters squared. School socioeconomic ranking, reflecting the proportion of children enrolled from families with higher socioeconomic status,[3-6] was obtained from the Guangdong Provincial Department of Education.[7]

To better estimate effects caused by potential confounding factors at the individual level, we randomly sampled 28 among the 110 schools, and then sampled 25% of students per grade in each school, for a total subset of 8,400 students. The response rate of this cohort to an additional set of questionnaires, detailing risk factors for myopia, including parental myopia and mean daily time spent on screen use, reading, and outdoor activities after school time, was 95.2%.[8-11] The average outdoor activity time during the school semester days (T_{School}) was calculated using the time spent outdoors on weekdays (T_{wd}) and on weekends (T_{we}): $T_{school} = (T_{wd} \times 5+T_{we} \times 2) \div 7$. Average outdoor activity time during the weekends (T_{wd}) was used as a proxy for time spent outdoors during the summer and winter holidays (3 months per school year). Therefore, the average outdoor activity time during the school years (T_{year}) was calculated as: $T_{year} = [(T_{school} \times 9) + (T_{we} \times 3)] \div 12$.

© 2024 Yang Y et al. *JAMA Ophthalmology*.

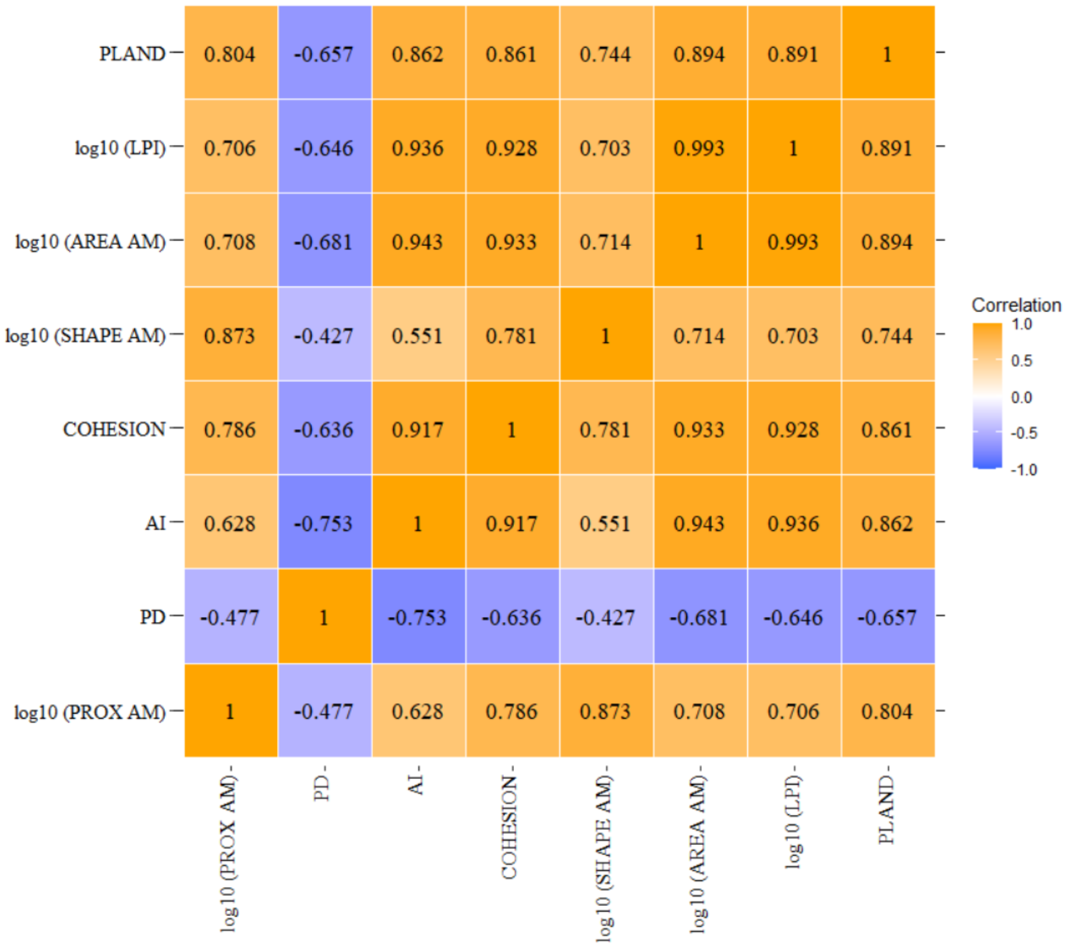

eFigure 1. Heatmap of the Correlation Matrix of Landscape Metrics

PROX_AM: area-weighted mean of proximity index; AI: aggregation index; PD: patch density; COHESION: cohesion index; PLAND: percentage of landscape; LPI: largest patch index; AREA_AM: area-weighted mean greenness area; SHAPE_AM: area-weighted mean patch shape index.

© 2024 Yang Y et al. *JAMA Ophthalmology.*

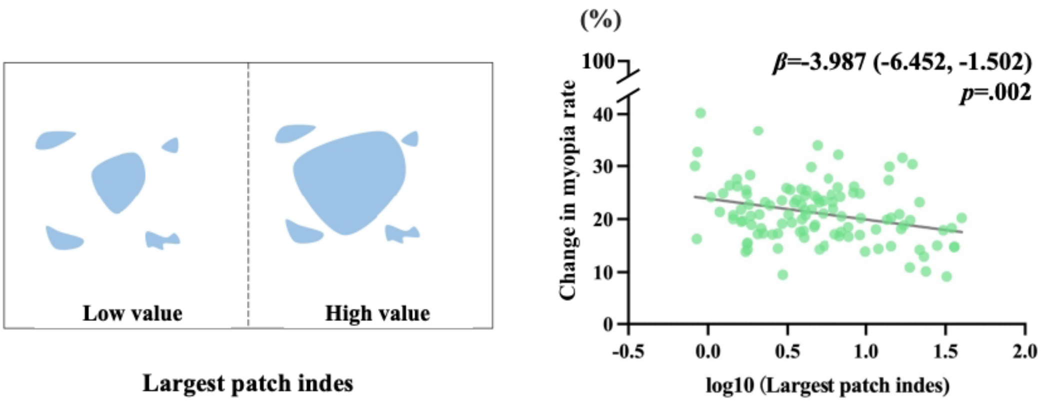

eFigure 2. Association Between Myopia and Largest Patch Index (LPI)

A higher value of LPI indicates a higher percentage occupied by the largest patch of green space out of the total area (value range: 0%<LPI≤100%). A 10% increase in LPI was associated with a 0.4% decrease in myopia rate (95% CI: -0.6% to -0.2%, p=.002).

© 2024 Yang Y et al. *JAMA Ophthalmology.*

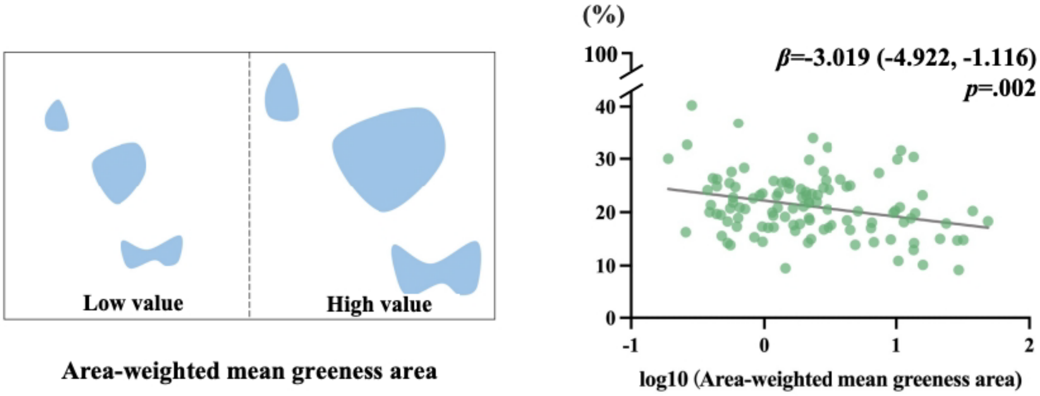

eFigure 3. Association Between Myopia and Area-Weighted Mean of Greenness Area (AREA_AM)

A higher value of AREA_AM indicates a larger area-weighted mean green space (value range: AREA_AM>0 m^2). A 10% increase in AREA_AM was associated with a smaller increase in myopia rate (-0.3%, 95% CI: -0.5% to -0.1%, p=.002).

© 2024 Yang Y et al. *JAMA Ophthalmology.*

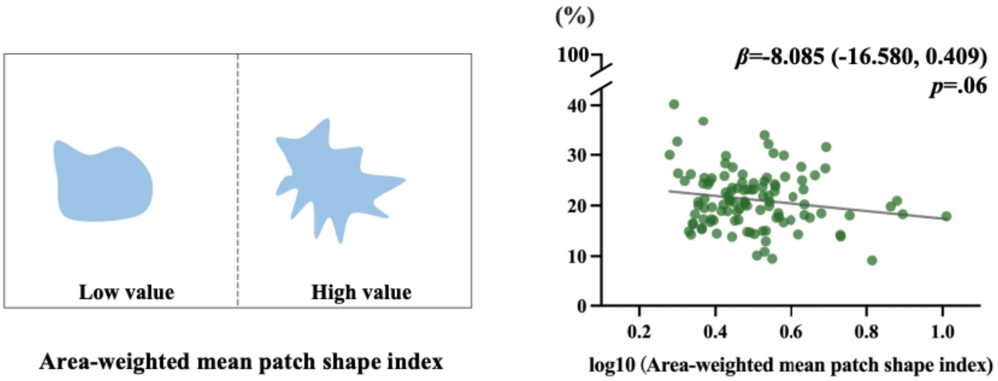

eFigure 4. Association Between Myopia and Area-Weighted Mean Shape Index (SHAPE_AM)

A higher value of SHAPE_AM indicates a more irregular shape of green space (value range: SHAPE_AM≥1). SHAPE_AM was not associated with the changes in myopia rate (-0.08, 95% CI: -0.2 to 0.04, p=.06)

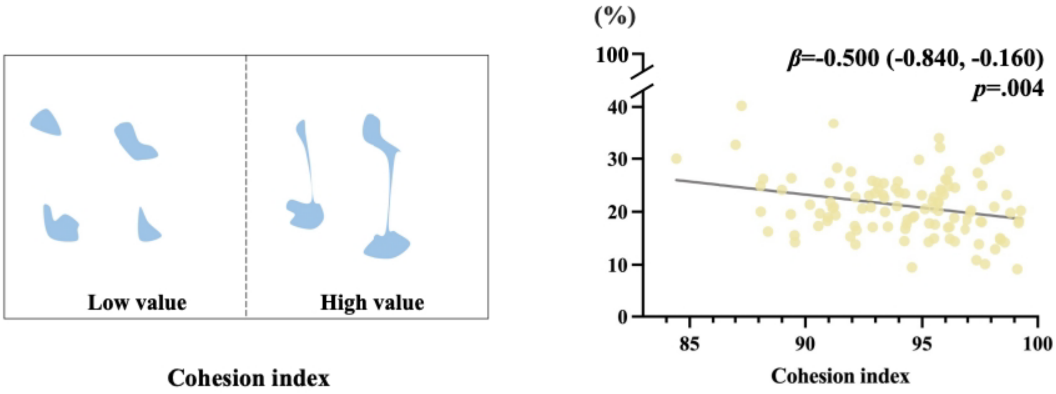

eFigure 5. Association Between Myopia and Cohesion Index (COHESION)

A higher value of COHESION indicates a more connected green space pattern (value range: 0<COHESION<100). The increase in COHESION was associated with a lower change in school myopia rate (-0.5%, 95% CI: -0.8% to -0.2%, p=.004).

© 2024 Yang Y et al. *JAMA Ophthalmology*.

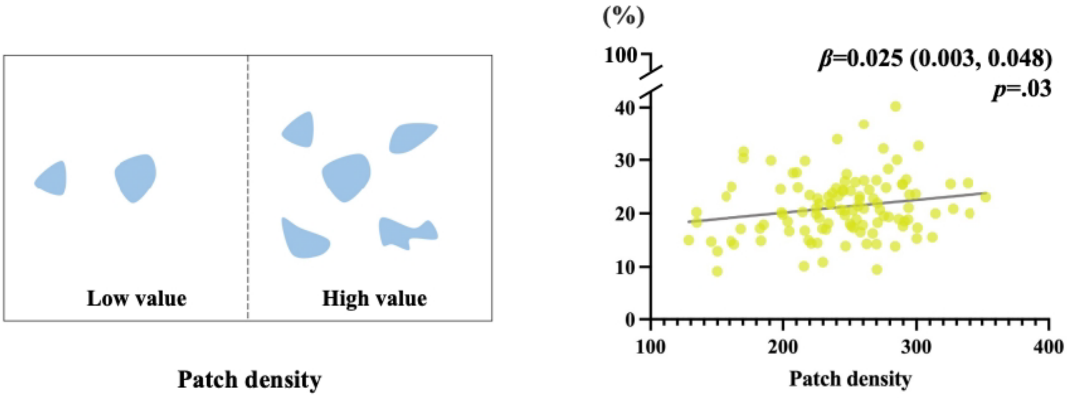

eFigure 6. Association Between Myopia and Patch Density (PD)

A higher value of PD indicates a more fragmented area of green space (value range: PD≥0). PD was positively associated with the change in school myopia rate (0.03%, 95% CI: -0.003% to -0.05%, p=.03).

© 2024 Yang Y et al. *JAMA Ophthalmology*.

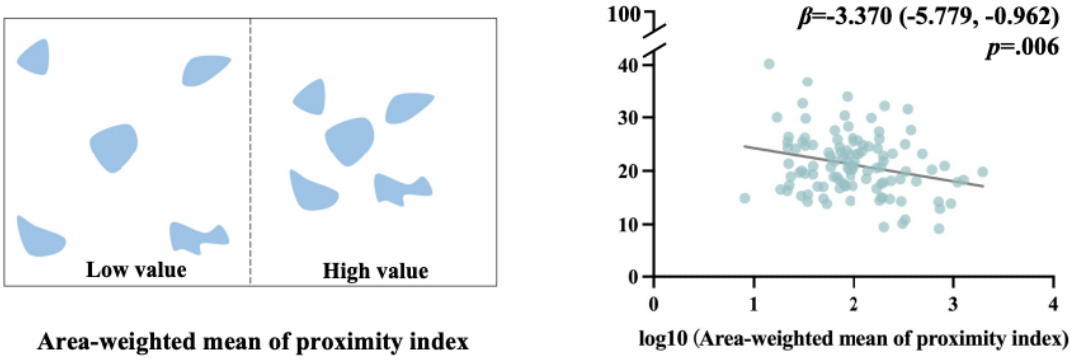

eFigure 7. Association Between Myopia and Proximity Index (PROX_AM)

A higher area-weighted mean of PROX_AM indicates a smaller distance with a less fragmented distribution of green patches within a campus buffer zone (value range: PROX_AM≥0). A 10% increase in PROX_AM was associated with a smaller increase in myopia rate (-0.3%, 95% CI: -0.6% to -1.0%, p=.007).

© 2024 Yang Y et al. *JAMA Ophthalmology.*

eTable 1. Characteristics of the Landscape Metrics and Population of the Studied Schools

Variables	N	Mean	SD
**Landscape metrics**			
PLAND	110	22.929	10.667
log 10 (LPI)	110	0.687	0.427
log 10 (AREA_AM)	110	0.338	0.558
log 10 (SHAPE_AM)	110	0.501	0.136
AI	110	83.400	5.505
COHESION	110	94.237	3.183
PD	110	242.802	47.730
log 10 (PROX_AM)	110	2.001	0.471
**Myopia variables**			
Myopia rate at baseline	110	0.119	0.037
Mean SER at baseline, diopter	110	-0.170	0.066
**Demographic variables**			
Percentage of boys, %	110	56.2	2.3
Mean age, years	110	7.404	0.264
Student-density	110	0.175	0.220

N: number;
SD, standard deviation;
PLAND: percentage of landscape;
AI: aggregation index;
COHESION: cohesion index;
LPI: largest patch index;
AREA_AM: area-weighted mean greenness area;
SHAPE_AM: area-weighted mean patch shape index;
PD: patch density;
PROX_AM: area-weighted mean of proximity index;
SER: spherical equivalent refraction.

© 2024 Yang Y et al. *JAMA Ophthalmology*.

eTable 2. Sensitivity Analyses of the Association Between Myopia-Related Green Space Morphology Index and School-Level Increase in Myopia Rate Using the Linear Regression Model

**Definition of myopia**	**SER ≤ −0.75 diopters**	
**Variables**	**Unstandardized β (95% CI)**	***p*-value**
PLAND	-0.001 (-0.002, -0.0003)	.01
log 10 (LPI)	-0.033 (-0.054, -0.011)	.004
log 10 (AREA _AM)	-0.030 (-0.049, -0.011)	.002
log 10 (SHAPE_AM)	-0.064 (-0.140, 0.012)	.10
AI	-0.002 (-0.004, -0.0004)	.01
COHESION	-0.003 (-0.007, -0.001)	.02
PD	0.0002 (-0.000008, 0.0004)	.06
log 10 (PROX_AM)	0.030 (-0.051, 0.008)	.008

N=110;
PLAND: percentage of landscape;
AI: aggregation index;
COHESION: cohesion index;
LPI: largest patch index;
AREA_AM: area-weighted mean greenness area;
SHAPE_AM: area-weighted mean patch shape index;
PD: patch density;
PROX_AM: area-weighted mean of proximity index;
SER: spherical equivalent refraction.

© 2024 Yang Y et al. *JAMA Ophthalmology*.

eTable 3. Association Between Myopia-Related Green Space Morphology Index and School-Level Increase in Myopia Rate Using the Linear Regression Model

**Variables**	**Unstandardized β (95% CI)**	***p*-value**
**Myopia-related green space morphology index**	-0.017 (-0.027, -0.006)	.002
**Mean age, years**	0.028 (-0.021, 0.077)	.26
**Percentage of boys, %**	-0.005 (-0.475, 0.466)	.98
**Student-density**	0.015 (-0.034, 0.064)	.55
**School socioeconomic ranking**		
Highest	-0.004 (-0.033, 0.026)	.03
Moderate	-0.005 (-0.030, 0.019)	.02
Lowest	1	
**Myopia rate at baseline**	-0.213 (-0.558, 0.133)	.23

N=110

© 2024 Yang Y et al. *JAMA Ophthalmology*.

eTable 4. Association Between Myopia-Related Green Space Morphology Index and Myopia Incidence at the Individual-Level Among Students Without Myopia Who Were Followed Using the Mixed-Effects Logistic Regression Model

**Variables**	**Adjusted OR (95% CI)**	***p*-value**
**Myopia-related green space morphology index**	0.902 (0.849, 0.959)	<.001
**Age, years**	1.470 (1.451, 1.490)	<.001
**Boy sex**	0.681 (0.661, 0.701)	<.001
**Student-density**	1.054 (0.794, 1.398)	.71
**School socioeconomic ranking**		
Highest	0.956 (0.807, 1.132)	.51
Moderate	0.951 (0.828, 1.093)	.48
Lowest	1	
**SER at baseline, diopter**	0.170 (0.149, 0.195)	<.001

N=101,897;
The school clustering effect was taken into account;
SER: spherical equivalent refraction.

© 2024 Yang Y et al. *JAMA Ophthalmology*.

eTable 5. Association Between Myopia-Related Green Space Morphology Index and Myopia Incidence at the Individual-Level Among the Subset Without Myopia Who Completed Questionnaire Using the Mixed-Effects Logistic Regression Model

Variables	Adjusted OR (95% CI)	*p*-value
**Myopia-related green space morphology index**	0.882 (0.804, 0.969)	.009
**Age, years**	1.455 (1.375, 1.540)	<.001
**Boy sex**	0.688 (0.611, 0.773)	<.001
**Student-density**	1.739 (0.181, 16.674)	.37
**School socioeconomic ranking**		
Highest	0.897 (0.707, 1.137)	.37
Moderate	0.941 (0.713, 1.242)	.67
Lowest	1	
**SER at baseline, diopter**	0.167 (0.097, 0.289)	<.001
**Father**		
With myopia	1.667 (1.480, 1.878)	<.001
**Mother**		
With myopia	1.644 (1.459, 1.853)	<.001
**Average reading time**		
<1 h	1	
1-2 h	1.006 (0.866, 1.169)	.93
2-3 h	1.138 (0.951, 1.362)	.16
3-4 h	0.927 (0.697, 1.233)	.60
>4 h	1.703 (1.216, 2.383)	.002
**Average screen time**		
<1 h	1	
1-2 h	1.985 (1.659, 2.375)	<.001
>2 h	3.051 (2.376, 3.919)	<.001
**Average outdoor activity time**		
<1 h	1	
1-2 h	0.979 (0.864, 1.110)	.74
>2 h	0.542 (0.440, 0.669)	<.001

N=7,006;
The cluster effects within school were taken into account;
SER: spherical equivalent refraction.

© 2024 Yang Y et al. *JAMA Ophthalmology*.

eReferences.

1. Jia P, Stein A, James P, et al. Earth observation: investigating noncommunicable diseases from space. *Annual review of public health* 2019; **40**: 85-104.

2. Hyman L, Gwiazda J, Hussein M, et al. Relationship of age, sex, and ethnicity with myopia progression and axial elongation in the correction of myopia evaluation trial. *Archives of ophthalmology* 2005; **123**(7): 977-87.

3. Wen D. school choices in compulsory education stage and its impact on vulnerable groups in China's urban areas. *Peking University Education Review* 2006; **4**(2): 12-23.

4. Chen Y, Fang C. Social stratification and education divide--an empirical study on the equity of institutional arrangement of "nearest admission to schools by district" during the compulsory education stage. *Jiangsu Social Science* 2007; **28**(1): 229-35.

5. Li X. Study on school choice behaviour and equality of distribution of educational opportunities at the stage of compulsory education: An empirical analysis of family education expenditure of 18 cities in China. *Education Research* 2008; **30**(3): 67-72.

6. Wu Y. Educational division system and educational stratification in China (1978-2008). *Sociological Study* 2013; **43**(4): 179-202.

7. EDoG P. Guangdong Province Primary School Evaluation and Management Measures. *http://jyjgzgovcn/yw2/zcfg/content/post_2584220html* 2009.

8. Jones LA, Sinnott LT, Mutti DO, et al. Parental history of myopia, sports and outdoor activities, and future myopia. *Investigative ophthalmology & visual science* 2007;**48**(8):3524-32.

9. Saxena R, Vashist P, Tandon R, et al. Prevalence of myopia and its risk factors in urban school children in Delhi: the North India Myopia Study (NIM Study). *PloS one* 2015;**10**(2).

10. Ip JM, Saw S-M, Rose KA, et al. Role of near work in myopia: findings in a sample of Australian school children. Investigative ophthalmology & visual science 2008; **49**(7): 2903-10.

© 2024 Yang Y et al. *JAMA Ophthalmology.*

11. He M, Xiang F, Zeng Y, et al. Effect of time spent outdoors at school on the development of myopia among children in China: a randomized clinical trial. Jama 2015; **314**(11): 1142-8.

© 2024 Yang Y et al. *JAMA Ophthalmology*.
