# Supplementary material for: Green Space Morphology and School Myopia in China
Source: JAMA Ophthalmol. 2024 Jan 4;142(2):115–22. doi: 10.1001/jamaophthalmol.2023.6015 (PMC10767644; doi:10.1001/jamaophthalmol.2023.6015)
Supplement: Supplement 2. — Data Sharing Statement [file jamaophthalmol-e236015-s002.pdf]

Data Sharing Statement

Yang. Green Space Morphology and School Myopia in China. *JAMA Ophthalmol*. Published January 04, 2024. doi:10.1001/jamaophthalmol.2023.6015

Data
Data available: No
